# *catena*-Poly[[dipyridine­cadmium(II)]-*μ*-5-amino-2,4,6-triiodo­isophthalato]

**DOI:** 10.1107/S1600536810039498

**Published:** 2010-10-09

**Authors:** Yang Zou

**Affiliations:** a Chemistry Department, Zhejiang Sci-Tech University, Hangzhou, 310018, People’s Republic of China

## Abstract

The hydro­thermal reaction of cadmium(II) nitrate with 5-amino-2,4,6-triiodo­isophthalic acid and pyridine in DMF solution leads to the formation of the title compound, [Cd(C_8_H_2_I_3_NO_4_)(C_5_H_5_N)_2_]_*n*_. The structure contains a four-coordinate Cd^2+ ^ion in a distorted tetra­hedral geometry, which lies on a crystallographic twofold rotation axis. The Cd^2+ ^ion is bonded to two N atoms from two pyridine ligands and two carboxyl­ate O atoms from two 5-amino-2,4,6-triiodo­isophthalate anions. The Cd—O distances are 2.429 (5) and 2.305 (5) Å and the Cd—N distance is 2.236 (8) Å. The two carboxyl­ate groups of individual 5-amino-2,4,6-triiodo­isophthalate anions act as a bridge to the Cd^2+^ atoms. leading to a chain structure along the *c* axis.

## Related literature

For the isotypic Hg complex, see: Zhang *et al.* (2008 ▶). For the structure of 5-amino-2,4,6-triiodo­isophthalic acid monohydrate, see: Beck & Sheldrick (2008 ▶). For the structures of related metal complexes, see: Dai *et al.* (2008 ▶)·For the use of triiodinated aromatic compounds in radiology, see: Estep *et al.* (2000 ▶).
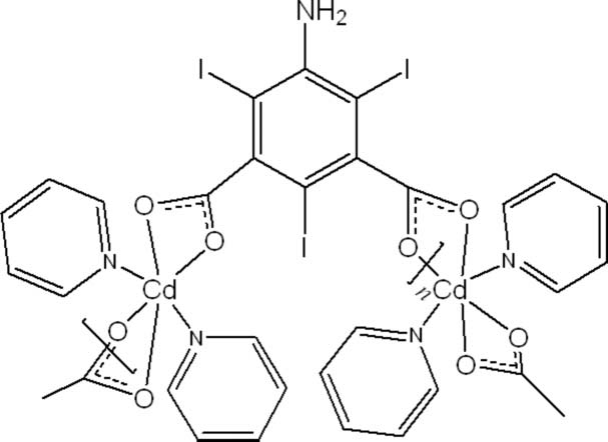


## Experimental

### 

#### Crystal data

[Cd(C_8_H_2_I_3_NO_4_)(C_5_H_5_N)_2_]
*M* *_r_* = 827.41
Tetragonal, 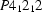

*a* = 11.824 (3) Å
*c* = 15.841 (9) Å
*V* = 2214.7 (15) Å^3^
*Z* = 4
Mo *K*α radiation
μ = 5.20 mm^−1^
*T* = 293 K
0.25 × 0.25 × 0.20 mm

#### Data collection

Bruker SMART CCD diffractometer
Absorption correction: multi-scan (*SADABS*; Bruker 2003 ▶) *T*_min_ = 0.357, *T*_max_ = 0.423
14248 measured reflections
2714 independent reflections
1949 reflections with *I* > 2σ(*I*)
*R*_int_ = 0.054

#### Refinement

*R*[*F* ^2^ > 2σ(*F* ^2^)] = 0.038
*wR*(*F* ^2^) = 0.089
*S* = 1.02
2714 reflections
134 parameters
60 restraints
H-atom parameters constrained
Δρ_max_ = 0.60 e Å^−3^
Δρ_min_ = −0.87 e Å^−3^
Absolute structure: Flack (1983 ▶), 1096 Friedel pairs
Flack parameter: −0.04 (6)

### 

Data collection: *SMART* (Bruker, 2003 ▶); cell refinement: *SAINT* (Bruker, 2003 ▶); data reduction: *SAINT*; program(s) used to solve structure: *SHELXS97* (Sheldrick, 2008 ▶); program(s) used to refine structure: *SHELXL97* (Sheldrick, 2008 ▶); molecular graphics: *XP* in *SHELXTL* (Sheldrick, 2008 ▶) and *DIAMOND* (Brandenburg, 2000 ▶); software used to prepare material for publication: *SHELXTL* and *PLATON* (Spek, 2009 ▶).

## Supplementary Material

Crystal structure: contains datablocks I, New_Global_Publ_Block. DOI: 10.1107/S1600536810039498/si2296sup1.cif

Structure factors: contains datablocks I. DOI: 10.1107/S1600536810039498/si2296Isup2.hkl

Additional supplementary materials:  crystallographic information; 3D view; checkCIF report

## Figures and Tables

**Fig. 1.**
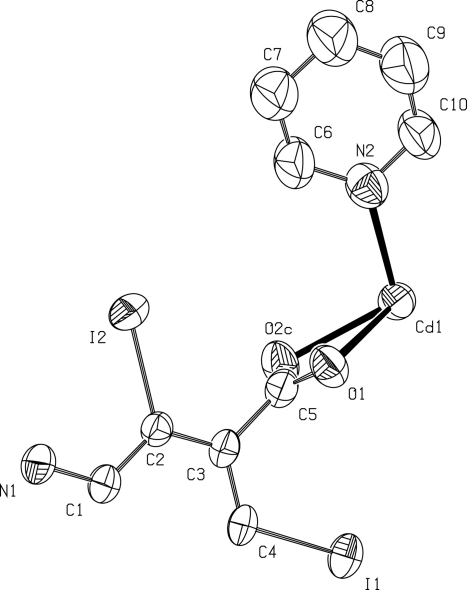
ORTEP plot of the title complex with atom numbering scheme. Thermal ellipsoids are drawn at 40% probability level.

**Fig. 2.**
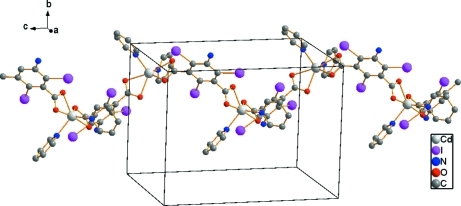
A section of the infinite [Cd(ATIA)(pyr)2]n chain along the c axis.

## supplementary crystallographic information

### Comment

5-Amino-2,4,6-triiodoisophthalic acid (ATIA), is the precursor and core structure of triiodinated contrast media used in radiology (Estep *et al.*, 2000). The crystal structure of this compound was reported recently (Beck *et al.*, 2008), however, there are very few studies that have been reported on the structural characterization of its metal complexes (Dai *et al.*, 2008; Zhang *et al.*, 2008). Here we report the synthesis and crystal structure of the title complex *catena*-[bis(pyridine)-µ-5-amino-2,4,6-triiodoisophthalic acid-O,*O*-cadmium(II)].

In the title complex the central cadmium ion is coordinated by two nitrogen atoms from two pyridine ligands and two oxygen atoms from different ATIA ligands in a tetrahedral geometry. The bond lengths are 2.236 (8)Å for Cd1—N2; 2.305 (5) Å for Cd1—O2 and 2.429 (5) Å for Cd1—O1. Both carboxylate groups of ATIA ligand are deprotonated during the reaction, and the whole ligand acts as a bridging linker to connect two cadmium ions. Thus, the [Cd(pyr)_2_] units are infinitely connected by ATIA ligands along the *c* axis to give rise to a one-dimensional chain structure.

### Experimental

5-amino-2,4,6-triiodoisophthalic acid (0.5 mmol) was dissolved in 10 ml DMA, in which Cd(NO_3_)_2_(0.5 mmol) and 20 *µ*l pyridine were added in. The mixture was sealed in a Pyrex tube and heated at 358 K for 3 d. After cooling to room temperature, light yellow block crystals were obtained.

### Refinement

All H atoms were positioned geometrically and constrained as riding atoms, with C—H distance of 0.93 Å and *U*_iso_(H) set to 1.2 *U*_eq_(C) of the parent atom.

### Crystal data

**Table:**

[Cd(C_8_H_2_I_3_NO_4_)(C_5_H_5_N)_2_]	*D*_x_ = 2.482 Mg m^−^^3^
*M**_r_* = 827.41	Mo *K*α radiation, λ = 0.71073 Å
Tetragonal, *P*4_1_2_1_2	Cell parameters from 1949 reflections
Hall symbol: P 4abw 2nw	θ = 2.2–28.2°
*a* = 11.824 (3) Å	µ = 5.20 mm^−^^1^
*c* = 15.841 (9) Å	*T* = 293 K
*V* = 2214.7 (15) Å^3^	Block, light yellow
*Z* = 4	0.25 × 0.25 × 0.20 mm
*F*(000) = 1520	

### Data collection

**Table:**

Bruker SMART CCD diffractometer	2714 independent reflections
Radiation source: fine-focus sealed tube	1949 reflections with *I* > 2σ(*I*)
graphite	*R*_int_ = 0.054
Detector resolution: none pixels mm^-1^	θ_max_ = 28.2°, θ_min_ = 2.2°
phi and ω scans	*h* = −15→15
Absorption correction: multi-scan (*SADABS*; Bruker 2003)	*k* = −14→15
*T*_min_ = 0.357, *T*_max_ = 0.423	*l* = −17→21
14248 measured reflections	

### Refinement

**Table:**

Refinement on *F*^2^	Secondary atom site location: difference Fourier map
Least-squares matrix: full	Hydrogen site location: inferred from neighbouring sites
*R*[*F*^2^ > 2σ(*F*^2^)] = 0.038	H-atom parameters constrained
*wR*(*F*^2^) = 0.089	*w* = 1/[σ^2^(*F*_o_^2^) + (0.0338*P*)^2^ + 2.974*P*] where *P* = (*F*_o_^2^ + 2*F*_c_^2^)/3
*S* = 1.02	(Δ/σ)_max_ < 0.001
2714 reflections	Δρ_max_ = 0.60 e Å^−^^3^
134 parameters	Δρ_min_ = −0.87 e Å^−^^3^
60 restraints	Absolute structure: Flack (1983), 1096 Friedel pairs
Primary atom site location: structure-invariant direct methods	Flack parameter: −0.04 (6)

### Special details

**Table:**

Geometry. All e.s.d.'s (except the e.s.d. in the dihedral angle between two l.s. planes) are estimated using the full covariance matrix. The cell e.s.d.'s are taken into account individually in the estimation of e.s.d.'s in distances, angles and torsion angles; correlations between e.s.d.'s in cell parameters are only used when they are defined by crystal symmetry. An approximate (isotropic) treatment of cell e.s.d.'s is used for estimating e.s.d.'s involving l.s. planes.
Refinement. Refinement of *F*^2^ against ALL reflections. The weighted *R*-factor *wR* and goodness of fit *S* are based on *F*^2^, conventional *R*-factors *R* are based on *F*, with *F* set to zero for negative *F*^2^. The threshold expression of *F*^2^ > σ(*F*^2^) is used only for calculating *R*-factors(gt) *etc*. and is not relevant to the choice of reflections for refinement. *R*-factors based on *F*^2^ are statistically about twice as large as those based on *F*, and *R*- factors based on ALL data will be even larger.

### Fractional atomic coordinates and isotropic or equivalent isotropic displacement parameters (Å^2^)

**Table:**

	*x*	*y*	*z*	*U*_iso_*/*U*_eq_	Occ. (<1)
Cd1	0.62771 (5)	0.37229 (5)	0.2500	0.0572 (2)	
I1	0.51631 (4)	0.51631 (4)	0.0000	0.0630 (2)	
I2	0.28105 (5)	0.12921 (6)	0.17206 (4)	0.0802 (2)	
N1	0.1416 (5)	0.1416 (5)	0.0000	0.064 (2)	
H1A	0.1357	0.0961	0.0421	0.077*	0.50
H1B	0.0961	0.1357	−0.0421	0.077*	0.50
N2	0.6179 (7)	0.2214 (7)	0.3348 (5)	0.0771 (19)	
O1	0.5507 (5)	0.2646 (5)	0.1328 (3)	0.0668 (15)	
O2	0.6079 (5)	0.5525 (4)	0.3035 (3)	0.0684 (16)	
C1	0.2221 (6)	0.2221 (6)	0.0000	0.051 (2)	
C2	0.2982 (6)	0.2357 (6)	0.0666 (4)	0.0462 (16)	
C3	0.3809 (6)	0.3171 (6)	0.0673 (4)	0.0479 (17)	
C4	0.3908 (5)	0.3908 (5)	0.0000	0.043 (2)	
C5	0.4652 (7)	0.3247 (7)	0.1375 (5)	0.0540 (19)	
C6	0.5209 (10)	0.1691 (10)	0.3519 (9)	0.113 (3)	
H6	0.4560	0.1946	0.3248	0.135*	
C7	0.5108 (12)	0.0835 (12)	0.4049 (10)	0.134 (4)	
H7	0.4419	0.0464	0.4114	0.161*	
C8	0.6063 (12)	0.0494 (13)	0.4514 (11)	0.147 (4)	
H8	0.6026	−0.0039	0.4944	0.177*	
C9	0.7027 (12)	0.0999 (13)	0.4288 (10)	0.143 (4)	
H9	0.7701	0.0745	0.4523	0.172*	
C10	0.7060 (10)	0.1827 (10)	0.3754 (8)	0.113 (4)	
H10	0.7755	0.2169	0.3654	0.135*	

### Atomic displacement parameters (Å^2^)

**Table:**

	*U*^11^	*U*^22^	*U*^33^	*U*^12^	*U*^13^	*U*^23^
Cd1	0.0617 (3)	0.0617 (3)	0.0482 (4)	−0.0120 (4)	−0.0049 (3)	−0.0049 (3)
I1	0.0559 (3)	0.0559 (3)	0.0773 (5)	−0.0148 (3)	0.0062 (3)	−0.0062 (3)
I2	0.0755 (4)	0.0957 (5)	0.0695 (3)	−0.0276 (4)	−0.0027 (3)	0.0291 (3)
N1	0.060 (4)	0.060 (4)	0.071 (5)	−0.027 (5)	−0.007 (4)	0.007 (4)
N2	0.075 (5)	0.073 (5)	0.083 (5)	−0.004 (4)	−0.013 (4)	−0.005 (4)
O1	0.054 (3)	0.091 (4)	0.056 (3)	0.008 (3)	−0.006 (3)	−0.012 (3)
O2	0.091 (4)	0.056 (3)	0.058 (3)	−0.003 (3)	−0.026 (3)	−0.005 (2)
C1	0.047 (3)	0.047 (3)	0.058 (5)	−0.009 (5)	0.003 (4)	−0.003 (4)
C2	0.041 (4)	0.050 (4)	0.048 (4)	−0.001 (3)	0.005 (3)	0.001 (3)
C3	0.040 (4)	0.052 (4)	0.052 (4)	−0.006 (3)	0.009 (3)	−0.005 (3)
C4	0.036 (3)	0.036 (3)	0.058 (5)	0.000 (4)	0.013 (3)	−0.013 (3)
C5	0.048 (5)	0.061 (5)	0.053 (4)	−0.011 (4)	0.006 (4)	−0.003 (4)
C6	0.077 (6)	0.105 (7)	0.156 (9)	−0.028 (6)	−0.026 (6)	0.040 (6)
C7	0.102 (7)	0.127 (8)	0.173 (9)	−0.027 (7)	−0.027 (7)	0.054 (7)
C8	0.105 (8)	0.142 (8)	0.196 (9)	−0.010 (7)	−0.031 (8)	0.062 (8)
C9	0.108 (8)	0.129 (8)	0.192 (9)	−0.026 (7)	−0.043 (8)	0.046 (8)
C10	0.075 (6)	0.102 (7)	0.161 (9)	−0.008 (6)	−0.038 (6)	0.048 (6)

### Geometric parameters (Å, °)

**Table:**

Cd1—N2	2.236 (8)	C1—C2	1.395 (9)
Cd1—N2^i^	2.236 (8)	C1—C2^ii^	1.395 (9)
Cd1—O2^i^	2.305 (5)	C2—C3	1.373 (9)
Cd1—O2	2.305 (5)	C3—C4	1.382 (8)
Cd1—O1^i^	2.429 (5)	C3—C5	1.495 (11)
Cd1—O1	2.429 (5)	C4—C3^ii^	1.382 (8)
Cd1—C5^i^	2.681 (8)	C5—O2^i^	1.246 (9)
Cd1—C5	2.681 (8)	C6—C7	1.321 (16)
I1—C4	2.098 (8)	C6—H6	0.9300
I2—C2	2.102 (7)	C7—C8	1.407 (17)
N1—C1	1.346 (12)	C7—H7	0.9300
N1—H1A	0.8600	C8—C9	1.336 (17)
N1—H1B	0.8600	C8—H8	0.9300
N2—C10	1.307 (12)	C9—C10	1.293 (16)
N2—C6	1.331 (13)	C9—H9	0.9300
O1—C5	1.239 (10)	C10—H10	0.9300
O2—C5^i^	1.246 (9)		
			
N2—Cd1—N2^i^	116.3 (4)	C5^i^—O2—Cd1	93.2 (5)
N2—Cd1—O2^i^	104.7 (3)	N1—C1—C2	122.5 (4)
N2^i^—Cd1—O2^i^	120.8 (2)	N1—C1—C2^ii^	122.5 (4)
N2—Cd1—O2	120.8 (2)	C2—C1—C2^ii^	115.0 (9)
N2^i^—Cd1—O2	104.7 (3)	C3—C2—C1	123.1 (7)
O2^i^—Cd1—O2	87.0 (3)	C3—C2—I2	118.8 (5)
N2—Cd1—O1^i^	82.4 (2)	C1—C2—I2	118.0 (5)
N2^i^—Cd1—O1^i^	91.2 (2)	C2—C3—C4	119.8 (7)
O2^i^—Cd1—O1^i^	136.7 (2)	C2—C3—C5	121.6 (7)
O2—Cd1—O1^i^	54.96 (18)	C4—C3—C5	118.6 (6)
N2—Cd1—O1	91.2 (2)	C3^ii^—C4—C3	119.2 (8)
N2^i^—Cd1—O1	82.4 (2)	C3^ii^—C4—I1	120.4 (4)
O2^i^—Cd1—O1	54.96 (18)	C3—C4—I1	120.4 (4)
O2—Cd1—O1	136.7 (2)	O1—C5—O2^i^	123.3 (7)
O1^i^—Cd1—O1	167.9 (3)	O1—C5—C3	117.7 (7)
N2—Cd1—C5^i^	100.6 (3)	O2^i^—C5—C3	119.0 (7)
N2^i^—Cd1—C5^i^	101.2 (3)	O1—C5—Cd1	64.8 (4)
O2^i^—Cd1—C5^i^	111.4 (2)	O2^i^—C5—Cd1	59.1 (4)
O2—Cd1—C5^i^	27.7 (2)	C3—C5—Cd1	169.9 (5)
O1^i^—Cd1—C5^i^	27.5 (2)	C7—C6—N2	124.3 (13)
O1—Cd1—C5^i^	164.3 (3)	C7—C6—H6	117.8
N2—Cd1—C5	101.2 (3)	N2—C6—H6	117.8
N2^i^—Cd1—C5	100.6 (3)	C6—C7—C8	118.7 (14)
O2^i^—Cd1—C5	27.7 (2)	C6—C7—H7	120.7
O2—Cd1—C5	111.4 (2)	C8—C7—H7	120.7
O1^i^—Cd1—C5	164.3 (3)	C9—C8—C7	114.6 (15)
O1—Cd1—C5	27.5 (2)	C9—C8—H8	122.7
C5^i^—Cd1—C5	138.0 (4)	C7—C8—H8	122.7
C1—N1—H1A	120.0	C10—C9—C8	122.6 (15)
C1—N1—H1B	120.0	C10—C9—H9	118.7
H1A—N1—H1B	120.0	C8—C9—H9	118.7
C10—N2—C6	115.1 (9)	C9—C10—N2	124.2 (12)
C10—N2—Cd1	122.3 (7)	C9—C10—H10	117.9
C6—N2—Cd1	122.5 (7)	N2—C10—H10	117.9
C5—O1—Cd1	87.7 (5)		

Symmetry codes: (i) −*y*+1, −*x*+1, −*z*+1/2; (ii) *y*, *x*, −*z*.

## References

[bb1] Beck, T. & Sheldrick, G. M. (2008). *Acta Cryst.* E**64**, o1286. 10.1107/S1600536808017741 PMC2961876 21202917

[bb2] Brandenburg, K. (2000). *DIAMOND* Crystal Impact GbR, Bonn, Germany.

[bb3] Bruker (2003). *SADABS*, *SAINT* and *SMART* Bruker AXS Inc., Madison, Wisconsin,USA.

[bb4] Dai, F., He, H. & Sun, D. (2008). *J. Am. Chem. Soc.* **130**, 14064–14065. 10.1021/ja805920t 18831586

[bb5] Estep, K. G., Josef, K. A., Bacon, E. R., Illig, C. R., Toner, J. L., Mishra, D., Blazak, W. F., Miller, D. M., Johnson, D. K., Allen, J. M., Spencer, A. & Wilson, S. A. (2000). *J. Med. Chem.* **43**, 1940–1948. 10.1021/jm990407i 10821706

[bb6] Flack, H. D. (1983). *Acta Cryst.* A**39**, 876–881.

[bb7] Sheldrick, G. M. (2008). *Acta Cryst.* A**64**, 112–122. 10.1107/S0108767307043930 18156677

[bb8] Spek, A. L. (2009). *Acta Cryst.* D**65**, 148–155. 10.1107/S090744490804362X PMC2631630 19171970

[bb9] Zhang, Y., Zhao, J., Tang, G. & Jiang, Z. (2008). *Acta Cryst.* E**64**, m1324. 10.1107/S1600536808030468 PMC2959462 21201059

